# Microstructural alterations measured by diffusion tensor imaging following transcatheter aortic valve replacement and their association with cerebral ischemic injury and cognitive function — a prospective study

**DOI:** 10.1007/s00234-022-03017-5

**Published:** 2022-08-01

**Authors:** Andrea Varga, Gyula Gyebnár, Ferenc Imre Suhai, Anikó Ilona Nagy, Lajos Rudolf Kozák, Csenge Ágnes Póka, Mirjam Franciska Turáni, Sarolta Borzsák, Astrid Apor, Andrea Bartykowszki, Bálint Szilveszter, Márton Kolossváry, Pál Maurovich-Horvat, Béla Merkely

**Affiliations:** 1 Department of Diagnostic Radiology, Heart and Vascular Centre, Semmelweis University, Budapest, Hungary; 2 Department of Interventional Radiology, Heart and Vascular Centre, Semmelweis University, Budapest, Hungary; 3 Medical Imaging Centre, Semmelweis University, Budapest, Hungary; 4 Department of Cardiology, Heart and Vascular Centre, Semmelweis University, Budapest, Hungary; 5 Department of Cardiology, Medical Centre - Hungarian Defence Forces, Budapest, Hungary; 6 MTA-SE Cardiovascular Imaging Research Group, Heart and Vascular Centre, Semmelweis University, Budapest, Hungary

**Keywords:** TAVR (transcatheter aortic valve replacement), MRI, DTI (diffusion tensor imaging), Cognitive function

## Abstract

**Purpose:**

We assessed diffusion tensor imaging (DTI) metric changes of the corpus callosum and cingulum correlated to postprocedural ischemic lesion load (ILL) and cognitive performance in transcatheter aortic valve replacement (TAVR).

**Methods:**

TAVR subjects had DTI post-TAVR (≤ 8 days) and at 6 months (78 participants, males 56%, age 78.8 years ± 6.3) and four neurocognitive tests (pre-TAVR, post-TAVR, 6 months, 1 year). DTI metrics (fractional anisotropy (FA), mean diffusivity (MD), axial diffusivity (AD), radial diffusivity (RD)) were calculated for 7 regions: corpus callosum (genu, body, splenium) and cingulum (cingulate gyrus, parahippocampal cingulum bilaterally). DTI metrics post-TAVR and at 6 months were compared with Student’s *t*-test (*p* < 0.0071) and ANOVA covarying for sex, ILL (*p* < 0.05) with post hoc analysis of ILL groups (*p* < 0.0167). Repeated-measures linear mixed-effect model (*p* < 0.05) was performed to investigate the effect of time and ILL on cognition.

**Results:**

At 6 months, significant decrease of the following DTI metrics was detected: AD (genu, body, splenium, right parahippocampal cingulum: *p* ≤ 0.0046); MD (body, both cingulate gyri: *p* ≤ 0.0050); RD (left cingulate gyrus: *p* = 0.0021); FA (splenium: *p* < 0.0001). ANOVA confirmed significant effect of female sex on AD + MD reduction (body, right cingulate gyrus) and AD reduction (left cingulate gyrus) (*p* ≤ 0.0254). Significant negative effect of ILL on some DTI metric changes was found (AD + MD-body: *p* ≤ 0.0050; MD-left cingulate gyrus: *p* = 0.0087).

Cognitive performance remained stable with significant negative correlation of ILL and retrograde memory and visual scores (*p* ≤ 0.0483).

**Conclusion:**

Significant effect of TAVR on cerebral microstructural integrity was found with reduced diffusivities opposite to the trends reported in various neurodegenerative conditions/ageing, notably in women and lower ILL, and with preserved/improved cognition.

Trial registration number.

NCT02826200 at ClinicalTrials.gov; date of registration: 07. July 2016.

**Supplementary Information:**

The online version contains supplementary material available at 10.1007/s00234-022-03017-5.

## Introduction

Transcatheter aortic valve replacement (TAVR) is a valid therapeutic option for patients with severe aortic stenosis (AS). TAVR had been first recommended in intermediate to high surgical risk or for inoperable patients [1] but recently has been approved in younger, lower-risk subjects [2].

Brain diffusion MRI refers to a group of MRI methods for non-invasive in vivo assessment of white matter (WM) integrity and connectivity, including diffusion tensor imaging (DTI). The change in different DTI-derived metrics reflects distinct aspects of WM microstructural alteration [3] and may help to quantify WM changes following TAVR.

The diffusion tensor is a 3 × 3 real, symmetric matrix representation of the three-dimensional water displacement probability in tissue. It can be represented as an ellipsoid with 3 main axes. The length of the longest half-axis reflects diffusion parallel to the supposed WM fibres, i.e. axial diffusivity (AD). The averaged lengths of the two shorter half-axes represent diffusivity perpendicular to the fibres; radial diffusivity (RD) [3, 4]. Fractional anisotropy (FA) is a measure of the directionality of diffusion within a voxel. FA values range between 0 (isotropic diffusion) and 1 (perfectly anisotropic diffusion). FA is the highest in compact fibre bundles oriented parallelly, such as the corpus callosum. Whereas myelin damage alone is associated with increased RD [5], subacute and chronic axonal damage with a combined myelin injury is shown to result in an increase in AD relative to RD and a decreased FA [6]. Decrease in FA is linked with loss of fibre integrity [7] and is observed in age-related WM changes [8–11] and various diseases [12, 13]. Mean diffusivity (MD) is the mean of the three ellipsoid half-axis lengths. MD reflects the magnitude of water diffusion and is proportional to the extracellular water content within a voxel, mainly used for detection of acute brain ischaemia [4]. Increase of diffusivities (MD, AD, RD) is also associated with ageing [9, 10, 14], mild cognitive impairment [15], cognitive impairment related to Alzheimer’ disease [16], Parkinson’s disease [17], type-2 diabetes [18] and multiple sclerosis [19].

The highly connected cingulum is an association tract, which plays an important role in cognition [20–22], so does the main interhemispheric connective tract, the corpus callosum [23].

TAVR is associated with a high incidence (up to 84%) of ischemic brain lesions (IBL) as detected by diffusion-weighted imaging [24–29]. The majority of these events did not manifest as overt stroke but has a potential to affect both the short and long term cognition, which was extensively studied since the introduction of TAVR [30–34]. No discernible difference was reported in long term global cognitive function up to 24 months after TAVR with a debated, modest, but statistically significant improvement in short-term cognitive performance on a whole-group level [35–37]. In a recent meta-analysis, subgroup analysis was performed for the different post-TAVR cognitive outcomes estimating improvement in 19% and impairment in 7%. They also found a strong inverse correlation between low pre-TAVR cognitive performance and post-TAVR cognitive improvement. TAVR might affect cognitive function in either way. Relieving the AS improves ejection fraction, cerebral perfusion and cognition [38]. On the contrary, TAVR subjects often suffer from postprocedural cerebral ischemic events, which has an adverse effect on cognition [39, 40]. Nonetheless, it has to be emphasized that these frail patient cohorts are characterised by several inherent cerebrovascular risk factors, which might contribute to their ischemic brain injury.

We aimed to (1) assess DTI metric changes from post-TAVR to 6 months in the corpus callosum and cingulum, (2) correlate these changes with postprocedural ischemic lesion load (ILL) and (3) evaluate the cognitive trajectory of subjects undergoing TAVR. Our hypothesis was that DTI can detect significant microstructural alterations in the brain of TAVR patients in conjunction with preserved or improving cognitive function, despite a high percentage of TAVR related IBLs.

## Methods

### Study participants

**Fig. 1:**
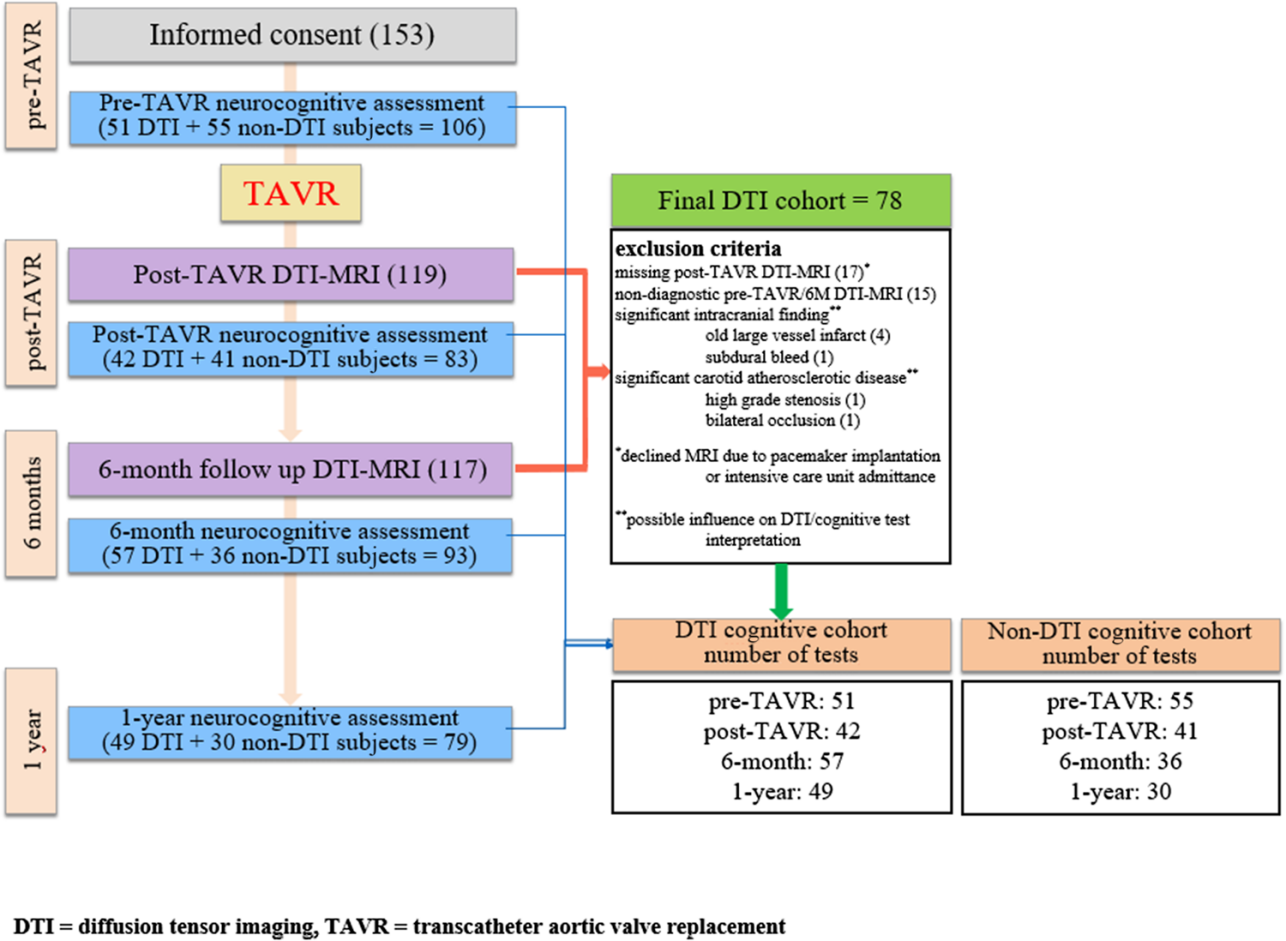
Flowchart of study design, diffusion tensor imaging and cognitive study cohorts

The *RulE out Transcatheter aORtic valve thrombosis with post-Implantation Computed tomography (RETORIC)* study was a prospective, single-centre, observational cohort study recruiting all TAVR subjects between November 2016 and June 2018 approved by the local ethics committee. Indication for TAVR was in concordance with the most recent guideline [1]. We included subjects into the DTI study with complete and adequate post-TAVR and 6M follow-up MRI datasets. The final DTI study cohort consisted of 78 participants. Inclusion criterion of the cognitive study was the accomplishment of the pre-TAVR baseline and at least one of the three further neurocognitive tests (divided into “DTI cognitive” and “non-DTI cognitive” cohorts) (Fig. 1). Written informed consent was obtained from all patients. The procedures used in this study adhere to the Declaration of Helsinki.

### MRI examination

The MRI examinations were performed on a 1.5 T MR scanner (Achieva1.5, Philips Medical Systems, Best, The Netherlands) using a 8-channel head coil at 2 timepoints: 1–8 days (mean 4 days) and 6 months ± 5 weeks (mean 28 weeks) after TAVR (referred to as post-TAVR and 6M follow-up MRI). Fluid-attenuated inversion recovery (FLAIR), T2-weighted, T2*-gradient echo and high-resolution 3D T1-weighted gradient echo sequences were obtained with diffusion MRI. The acquisition parameters are specified in Table 1.

**Table 1:** MRI protocol parameters

Sequence parameters	dMRI	FLAIR	T2W	T2*-GRE	3D T1W
Orientation	Axial	Axial	Axial	Axial	Sagittal
Repetition time (TR) (ms)	14–29	6000	3000–6460	890–1023	25
Echo time (TE) (ms)	62	120	100	23	4.6
Flip angle (FA) (degree)	90	90	90	18	30
Echo-planar imaging factor	59	-	-	-	-
Turbo factor	59	23	15	1	1
Inversion time (TI) (ms)	-	2000	-	-	-
Matrix	112 × 128	200 × 268	300 × 400	200 × 268	220 × 220
Field of view (FOV) (mm)	224–240	230	230	230	240
Number of excitations	1	2	2	2	1
slice thickness/spacing (mm)	2/2	4/5	4/5	4/5	1/1
Encoding directions	32	-	-	-	-
*b* values (s/mm^2^)	0 and 800	-	-	-	-

*dMRI*, diffusion MRI; *FLAIR*, fluid-attenuated inversion recovery; T2*-GRE, T2*-gradient echo

Diffusion MRI acquisitions were performed using a single-shot spin echo, echo-planar imaging sequence in 32 diffusion encoding directions with *b* = 800 s/mm^2^ and one *b* = 0 measurement. Whole brain coverage was obtained with 2-mm-thick contiguous axial slices. The total acquisition time was 7:30–8:30 min. From the diffusion MRI dataset, averaged diffusion-weighted images commonly referred to as “trace” and MD maps were automatically derived and used to calculate the ischemic lesion load (ILL).

### Image processing

Diffusion MRI data was processed in the Matlab-based ExploreDTI toolbox [41]. To correct distortions originating from patient motion, the diffusion weighted images (*b* = 800) were registered to the first *b* = 0 measurement volume. Patient motion and other distortions due to differences in magnetic susceptibility and those related to echo-planar imaging readout were corrected in one interpolation step using each subject’s 3D T1-weighted images as registration target [42]. Robust tensor fitting with outlier rejection was performed [43] and the average FA, MD, AD and RD values in ROIs covering major WM tracts were calculated on the corrected dMRI data.

### Identification of white matter regions of interest

The Johns Hopkins University WM tractography atlas was used for WM segmentation [44]. The atlas labels were transformed to each subject’s individual image space, for which ExploreDTI utilises the Elastix software [45].

**Fig. 2:**
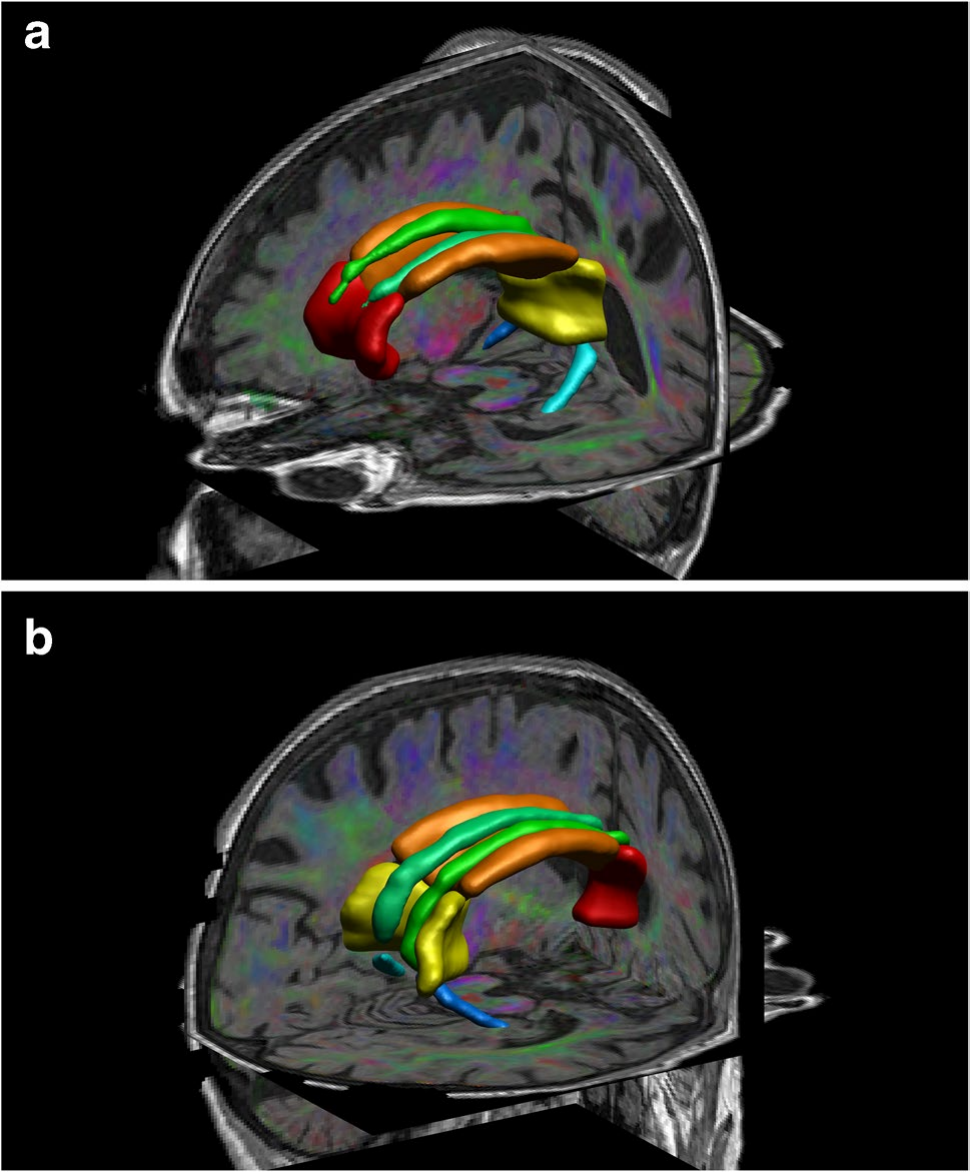
**a** 3D left anterior–posterior view; **b** 3D right posterior-anterior view of one study subject’s regions of interest projected over the fused colour coded fractional anisotropy and T1 weighted images. Red = genu of corpus callosum, orange = body of corpus callosum, yellow = splenium of corpus callosum, emerald green = right cingulate gyrus, jade green = left cingulate gyrus, dark blue = right parahippocampal cingulum, light blue = left parahippocampal cingulum

Seven WM ROIs were analysed: 3 callosal segments (genu, body, splenium of the corpus callosum) and 2 bilateral parts of the cingulum (right/left cingulate gyrus and right/left parahippocampal cingulum). These ROIs were subsequently corrected manually to avoid contamination from the grey matter or cerebrospinal fluid using the drawing functions of MRIcron (www.nitrc.org) by an experienced neuroradiologist (Fig. 2a and b).

### Ischemic lesion load calculation

The trace and MD images were visually inspected on an AGFA Picture Archiving and Communication System (PACS) workstation (Impax 6.5.2.657, Agfa HealthCare, Mortsel, Belgium). Two radiologists identified all lesions with restricted diffusion in consensus (i.e. high signal intensity on the “trace” images and corresponding low signal intensity on the MD map) and their number, localisation and volume were recorded. Lesion volume (mm^3^) was calculated as a product of the manually contoured surface (mm^2^) of each lesion and the slice thickness (2 mm). ILL was calculated as the sum of all lesion volumes.

Based on ILL, we created three groups of similar sample size as follows:

Group I: patients with *ILL* < 100 mm^3^ (31 subjects).

Group II: 100 mm^3^ < *ILL* < 300 mm^3^ (25 subjects).

Group III: 300 mm^3^ < ILL (22 subjects).

### Cognitive testing

The Hungarian repeatable version of Addenbrooke’s Cognitive Examination (ACE) was used for cognitive testing at four time points as follows:

i) 0–10 days (mean 2 days) prior TAVR (pre-TAVR test)
ii) 1–8 days (mean 4 days) after TAVR (post-TAVR test)
iii) 6 months ± 6 weeks (mean 28 weeks) after TAVR (6M test)
iv) 11–18 months (mean 14 months) after TAVR (1Y test)

Eight cognitive domains (orientation, concentration, evocation, anterograde memory, retrograde memory, verbal fluency, language and visuospatial processing) were assessed with summed up total ACE and mini mental state examination (MMSE) scores [46].

### Statistical analysis

To compare males and females in the DTI cohort we used two tailed, two sample *t*-test for continuous values; for categorical values the chi square test was applied (*p* < 0.05).

Student’s two tailed, paired sample *t*-test was used to assess differences between the averages of post-TAVR and 6M DTI scalar metrics (FA, AD, MD, AD) in seven WM ROIs. Bonferroni correction for 7 comparisons was employed; *p*-values < 0.0071 were considered significant.

The effects of age, sex and ILL on the DTI metric changes from post-TAVR to 6M were assessed using repeated measures analysis of variance (ANOVA) (*p* < 0.05) with post hoc tests between each pair of ILL groups in Matlab (MATLAB 8.3, The MathWorks Inc, Natick, MA, 2000). Regarding the three pairwise comparisons in the post hoc analysis, we applied Bonferroni correction and *p* < 0.0167 was considered significant. The increase or decrease of each DTI metric was appreciated with the help of box plots.

Spearman correlation test was performed to analyse the association of DTI metrics in each ROI and the cognitive results using Matlab (*p* < 0.0071).

We analysed the ACE and MMSE scores of the DTI study participants (DTI cognitive cohort) and those who underwent only cognitive testing without DTI (labelled as non-DTI cognitive cohort) with the Kruskal–Wallis test. We also compared the trends of all cognitive domains between the DTI and non-DTI groups using box plots.

To investigate the changes of cognitive scores over 1 year following TAVR, we used repeated-measures linear mixed-effect (LMM) model with a random intercept and random slope for time and first-order autoregressive within-group correlation structure fitted by maximising the restricted log-likelihood (R v4.0.5 Foundation for Statistical Computing, Vienna, Austria). The following variables were considered to have fixed effects: the pre-TAVR score of each cognitive domain, the ILL group (ILL group I + II vs ILL group III) and the time from TAVR in months.

Cognitive data were further analysed using the Wilcoxon signed-rank test to identify changes in performance across time points (pairwise comparisons of corresponding cognitive scores). The significance level was defined as *p* < 0.05.

## Results

### Patient characteristics

The age, gender, comorbidities and medical therapy of the 78 study subjects are reported in Table 2. Diabetes mellitus (55% vs 26%, *p* = 0.0128) and previous medical history of myocardial infarction (34% vs 6%, *p* = 0.0277) was significantly more frequent in males. The demographic data, other comorbidities, percentage of anticoagulant or antiplatelet therapy did not differ between the two genders.

**Table 2:** Age, gender, comorbidities and medical therapy of the 78 diffusion tensor imaging study subjects

***Demographics***	All: 78	Males	Females
Gender *N* (%)		44 (56)	34 (44)
Mean age ± SD (years)	78.8 ± 6.3	79 ± 6.2	78.5 ± 6.5
Body mass index ± SD (kg/m^2^)	27.4 ± 4.8	27.4 ± 3.6	27.4 ± 6.0
Body surface area ± SD (m^2^)	1.8 ± 0.2	1.9 ± 0.2	1.7 ± 0.2
CHA2DS2-VASc score	4.7 ± 1.3	4.5 ± 1.1	4.9 ± 1.6
***Co-morbidities***			
Hypertension *N* (%)	70 (90)	42 (95)	28 (82)
Diabetes mellitus type 2 N (%)	33 (42)	24 (55)*	9 (26)*
Hyperlipidemia *N* (%)	53 (70)	31 (70)	22 (62)
Current smoking *N* (%)	5 (6)	2 (5)	3 (9)
Former smoking *N* (%)	5 (6)	2 (5)	3 (9)
Atrial fibrillation/flutter *N* (%)	26 (33)	14 (32)	12 (35)
Previous TIA *N* (%)	2 (3)	1 (2)	1 (3)
Previous minor stroke *N* (%)	3 (4)	1 (2)	2 (6)
Coronary artery disease — post-myocardial infarction *N* (%)	17 (22)	15 (34)*	2 (6)*
Coronary artery disease — no myocardial infarction *N* (%)	18 (23)	12 (27)	6 (18)
Coronary revascularization — PCI *N* (%)	26 (33)	21 (48)	5 (15)
Coronary revascularization — CABG *N* (%)	5 (6)	5 (11)	0
Non-significant carotid artery stenosis *N* (%)	40 (51)	23 (52)	17 (50)
Previous carotid artery intervention *N* (%)	3 (4)	3 (7)	0
Heart failure NYHA II *N* (%)	40 (51)	23 (52)	17 (50)
Heart failure NYHA III *N* (%)	34 (44)	20 (45)	14 (41)
Heart failure NYHA IV *N* (%)	4 (5)	1 (2)	3 (9)
Liver disease *N* (%)	2 (3)	1 (2)	1 (3)
Any malignancy *N* (%)	15 (19)	11 (25)	4 (12)
Chronic obstructive pulmonary disease *N* (%)	13 (17)	5 (11)	8 (24)
Renal failure (*GFR* < 30) *N* (%)	3 (4)	2 (5)	1 (3)
***Medication***			
Anticoagulant therapy (vitamin K antagonist) *N* (%)	13 (17)	8 (18)	5 (15)
Anticoagulant therapy (NOAC) *N* (%)	10 (13)	8 (18)	2 (6)
Current antiplatelet therapy (single) *N* (%)	38 (49)	21 (48)	17 (50)
Current antiplatelet therapy (dual) *N* (%)	17 (22)	12 (27)	5 (15)
Statin therapy *N* (%)	50 (64)	29 (66)	21 (62)
ACE inhibitor/ARB *N* (%)	62 (80)	35 (80)	27 (79)
Beta blocker *N* (%)	66 (85)	38 (86)	28 (82)

*ACE*, angiotensin converting enzyme inhibitors; *ARB*, angiotensin II receptor blocker; *BMI*, body mass index; *CABG*, coronary artery bypass grafting; *CHA2DS2-VASc*, Congestive heart failure, Hypertension, Age, Diabetes, Stroke/TIA and VAScular disease score; *NOAC*, novel oral anticoagulant; *NYHA*, New York Heart association functional classification; *PCI*, percutaneous coronary intervention; *SD*, standard deviation. *Significant difference at *p* < 0.05

### Analysis of ischemic lesion load

Seventy-four out of 78 study subjects (95%) had recent ischemic lesions with restricted diffusion, 71 of them supratentorial (91%), most of them multiple (57/70, 81%) ranging in number from 2 to 20.

We considered infratentorial IBLs less relevant regarding cognitive function. A total of 363 supratentorial lesions were found, 194 in the left and 169 in the right cerebral hemisphere. The majority of these lesions were small (268/363, 74%, ≤ 5 mm; 109/363, 30%, measuring appr. 3 mm), mostly located at the cortical-subcortical interface. The median lesion volume was 38.9 mm^3^ (range: 14 mm^3^–17.4 cm^3^, interquartile range: 14–72.9 mm^3^). Three larger posterior cerebral artery territorial infarcts were detected in three subjects (measuring 7.2 cm^3^, 13.5 cm^3^ and 17.4 cm^3^; two on the left, another one on the right, without directly involving our ROIs). With special regard to ischemic injury in our ROIs, a part of the left parahippocampal cingulum was affected in one subject, and a small segment of the splenium was involved in another (both excluded from the ROIs during manual correction).

At 6 months, 7 out of 78 participants (9%) had de novo supratentorial IBL(s) with restricted diffusion: one lesion in 4 subjects, two lesions in 2, finally seven lesions in 1 subject. The ILL ranged from 14 to 232 mm^3^ (50% of the lesions were dot-like, measuring 3 mm).

### Analysis of DTI scalar metrics changes

Using the Student’s two tailed paired sample *t*-test with Bonferroni-correction, in 4 out of the 7 WM ROIs, significant reduction of AD was detected (genu and body: *p* < 0.0001; splenium and right parahippocampal cingulum: *p* ≤ 0.0046), coupled with significant decrease of the MD in the body of corpus callosum (*p* = 0.0012) and FA in the splenium (*p* < 0.0001). In the left cingulate gyrus, significant reduction of MD and RD was found (MD: *p* = 0.0008, RD: *p* = 0.0021) with significant MD reduction alone in the right cingulate gyrus: (MD: *p* = 0.0050) (Table 3). The other alterations were not significant.

**Table 3:** Two tailed paired *T*-test results with Bonferroni-correction of diffusion tensor metrics in seven white matter regions of interest

	DTI metrics	Mean (SD) post-TAVR	Mean (SD) 6-month follow-up	*p*-value	*T*
Body of corpus callosum	FA	0.5402 (0.0401)	0.5371 (0.0379)	0.1477	1.4623
	MD	1.2341 (0.0720)	1.2134 (0.0700)	**0.0012**	3.3586
	AD	2.0252 (0.0974)	1.9854 (0.1033)	** < 0.0001**	5.4712
	RD	0.8386 (0.0802)	0.8274 (0.0741)	0,0823	1.7604
Cingulate gyrus right	FA	0.4853 (0.0372)	0.4892 (0.0395)	0.2700	− 1.1113
	MD	0.8328 (0.0354'	0.8230 (0.0286)	**0.0050**	2.8961
	AD	1.3173 (0.0699)	1.3070 (0.0657)	0.1112	1.6112
	RD	0.5906 (0.0386)	0.5810 (0.0372)	0.0106	2.6212
Cingulate gyrus left	FA	0.5207 (0.0405)	0.5251 (0.0410)	0.2650	− 1.1229
	MD	0.8215 (0.0306)	0.8099 (0.0289)	**0.0008**	3.5074
	AD	1.3482 (0.0669)	1.3360 (0.0721)	0.0809	1.7685
	RD	0.5581 (0.0374)	0.5469 (0.0356)	**0.0021**	3.1907
Parahippocampal cingulum right	FA	0.4306 (0.0441)	0.4284 (0.0430)	0.6018	0.5239
	MD	0.8560 (0.0835)	0.8356 (0.0339)	0.0265	2.2615
	AD	1.2831 (0.1053)	1.2514 (0.0500)	**0.0045**	2.9265
	RD	0.6424 (0.0828)	0.6277 (0.0458)	0.1001	1.6643
Parahippocampal cingulum left	FA	0.4246 (0.0357)	0.4211 (0.0387)	0.4077	0.8325
	MD	0.8273 (0.0547)	0.8213 (0.0419)	0.3440	0.9522
	AD	1.2306 (0.0860)	1.2171 (0.0596)	0.1057	1.6369
	RD	0.6256 (0.0505)	0.6233 (0.0475)	0.7278	0.3493
Genu of corpus callosum	FA	0.5556 (0.0426)	0.5495 (0.0404)	0.0404	2.0853
	MD	1.1252 (0.0710)	1.1095 (0.0682)	0.0242	2.2989
	AD	1.8902 (0.1113)	1.8523 (0.1161)	** < 0.0001**	4.1680
	RD	0.7428 (0.0748)	0.7381 (0.0671)	0.4989	0.6795
Splenium of corpus callosum	FA	0.6404 (0.0373)	0.6305 (0.0338)	** < 0.0001**	4.2235
	MD	1.0249 (0.0687)	1.0269 (0.0640)	0.6882	− 0.4028
	AD	1.8682 (0.0923)	1.8504 (0.0886)	**0.0046**	2.9221
	RD	0.6032 (0.0729)	0.6152 (0.0662)	0.0289	− 2.2261

*AD*, axial diffusivity; *DTI*, diffusion tensor imaging; *FA*, fractional anisotropy; *MD*, mean diffusivity; *RD*, radial diffusivity; *SD*, standard deviation; *TAVR*, transcatheter aortic valve replacement. Note: Metric dimensions: *FA*, dimensionless; *MD*, *AD*, *RD*, 10^−4^ mm^2^/s

### Effects of sex and age on DTI metric changes

**Fig. 3:**
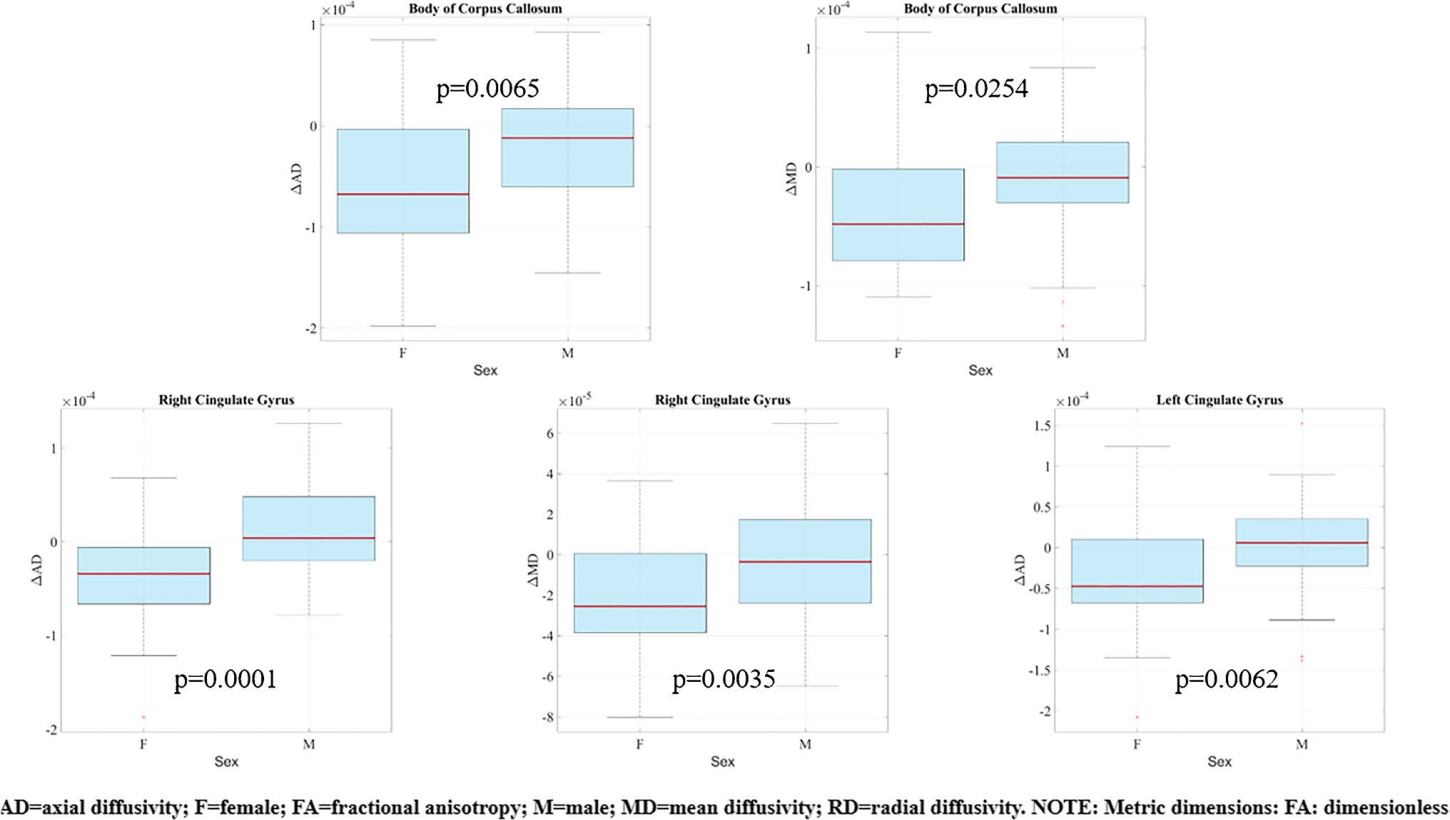
Box plots of significant associations of change in diffusion tensor imaging metrics with sex. Top row: change of axial diffusivity (left) and mean diffusivity (right) from post-TAVR to 6 months in the body of corpus callosum in females versus males. Bottom row: change of axial diffusivity (left) and mean diffusivity (middle) from baseline to 6 months in the right cingulate gyrus; change of axial diffusivity from baseline to 6 months in the left cingulate gyrus (right) in females versus males. (Red line = median; top of box = 25th percentile; bottom of box = 75th percentile; plotted whisker = the most extreme data value that is not an outlier; red cross = outlier)

The repeated measures ANOVA confirmed significant effect of female sex on AD/MD reduction. In women, significantly greater decrease of AD and MD was shown in the body of corpus callosum and in the right cingulate gyrus (body: AD – *p* = 0.0065, MD – *p* = 0.0254; right cingulate gyrus: AD – *p* = 0.0001, MD – *p* = 0.0035) with a significantly greater reduction of AD alone in the left cingulate gyrus (*p* = 0.0062) (Fig. 3 and Supplement Table 1).

No significant effect of age was detected in any of the DTI metric changes from post-TAVR to 6 M.

### Effects of ILL on DTI metric changes

**Fig. 4:**
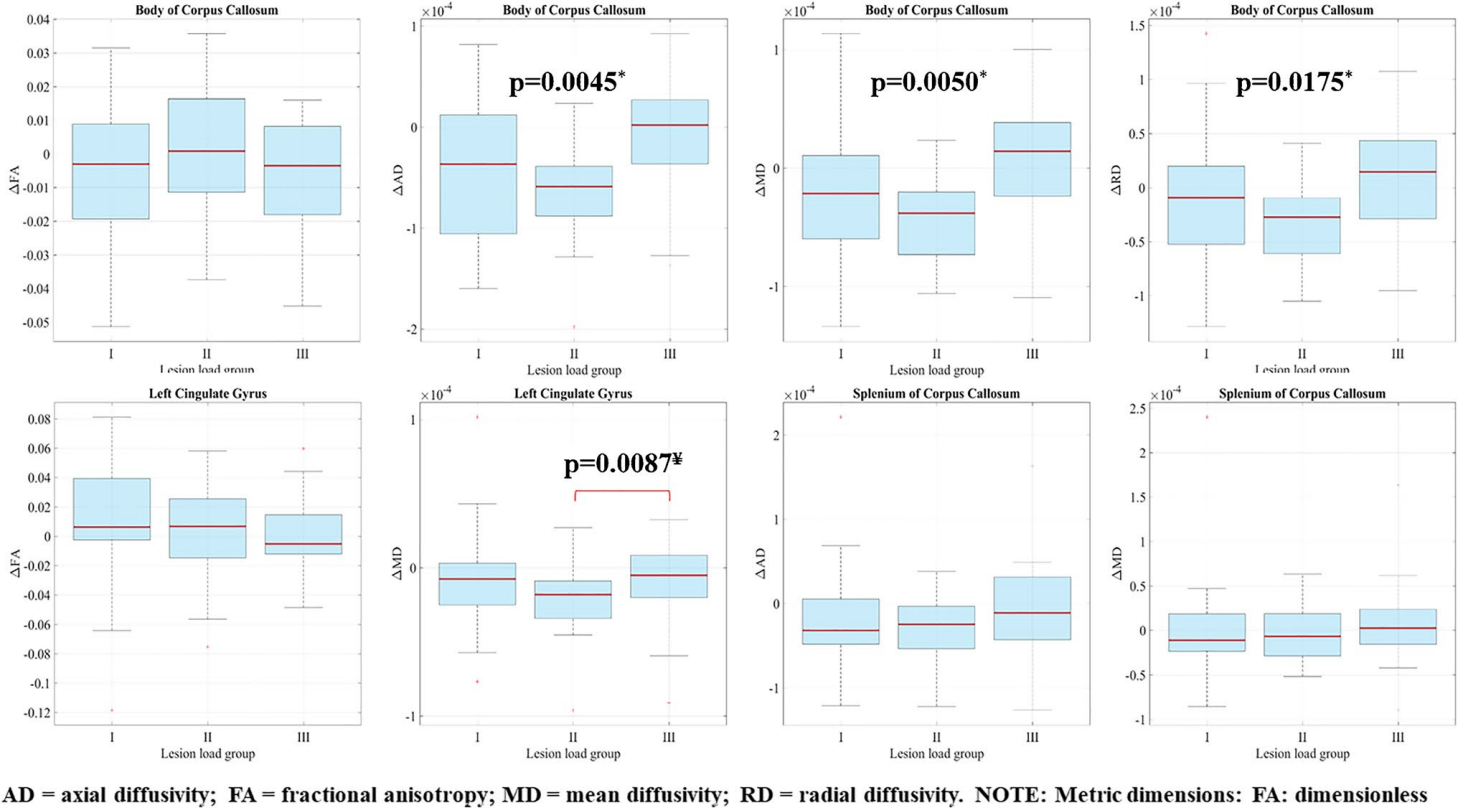
Box plots of association of change in diffusion tensior imaging metrics with ischemic lesion load (ILL). Top row (from left to right): change of fractional anisotropy, axial diffusivity, mean diffusivity and radial diffusivity from baseline to 6 months in the body of corpus callosum in ILL groups I, II and III. Bottom row (from left to right): change of fractional anisotropy and mean diffusivity from baseline to 6 months in the left cingulate gyrus; change of axial diffusivity and mean diffusivity from baseline to 6 months in the splenium of the corpus callosum in ILL groups I, II and III. (Red line = median; top of box = 25th percentile; bottom of box = 75th percentile; plotted whisker = the most extreme data value that is not an outlier; red cross = outlier). ^*^p-values from repeated measures analysis of variance across ILL groups. ^¥^*p*-value from post hoc analysis indicating significant between-group difference

The repeated measure ANOVA showed significant effect of ILL on certain DTI metric changes in 3 out of 7 ROIs (Fig. 4 and Supplement Table 1).

In the body of the corpus callosum, a significant within group effect of ILL was detected on the change of AD, MD and RD (*p* ≤ 0.0175 for all): in groups I–II, all the diffusivities decreased as opposed to the increase of diffusivities in group III. As revealed by the post hoc comparison, the difference was the most pronounced between the ILL group I and the ILL group III. The *p*-value for AD was 0.0183 (just above the Bonferroni-corrected significance level of *p* < 0.0167). The difference of MD did not reach the level of significance (*p* = 0.0457).

In the left cingulate gyrus, significant difference in MD reduction was found between the ILL group II and group III (*p* = 0.0087). The decrease of MD was more marked in the intermediate group relative to the group with the highest ILL.

In the splenium of the corpus callosum the mild increase of MD in ILL group III was in contrast to the reduction of MD in groups I and II, the difference was nevertheless non-significant with Bonferroni correction (*p* = 0.0462 for between-group comparison of I and III). The reduction of AD in ILL group III was less marked as compared to groups I-II (non-significant difference).

The other associations were non significant.

### Cognitive trajectory

The number of the completed cognitive tests were as follows (Fig. 1):

                                  Whole cohort               DTI cognitive cohort                non-DTI cognitive cohort

Pre-TAVR                  106                              51                                             55

Post-TAVR                 83                               42                                             41

6 months                    93                               57                                             36

1 year                         79                               49                                             30

As the Kruskal–Wallis test did not show a significant difference in the distribution of ACE and MMSE scores between the DTI and the non-DTI cognitive cohorts, and the corresponding box plots showed a very similar trend in all cognitive domains, therefore, we opted for analysing the cognitive performance of the whole study cohort.

**Fig. 5:**
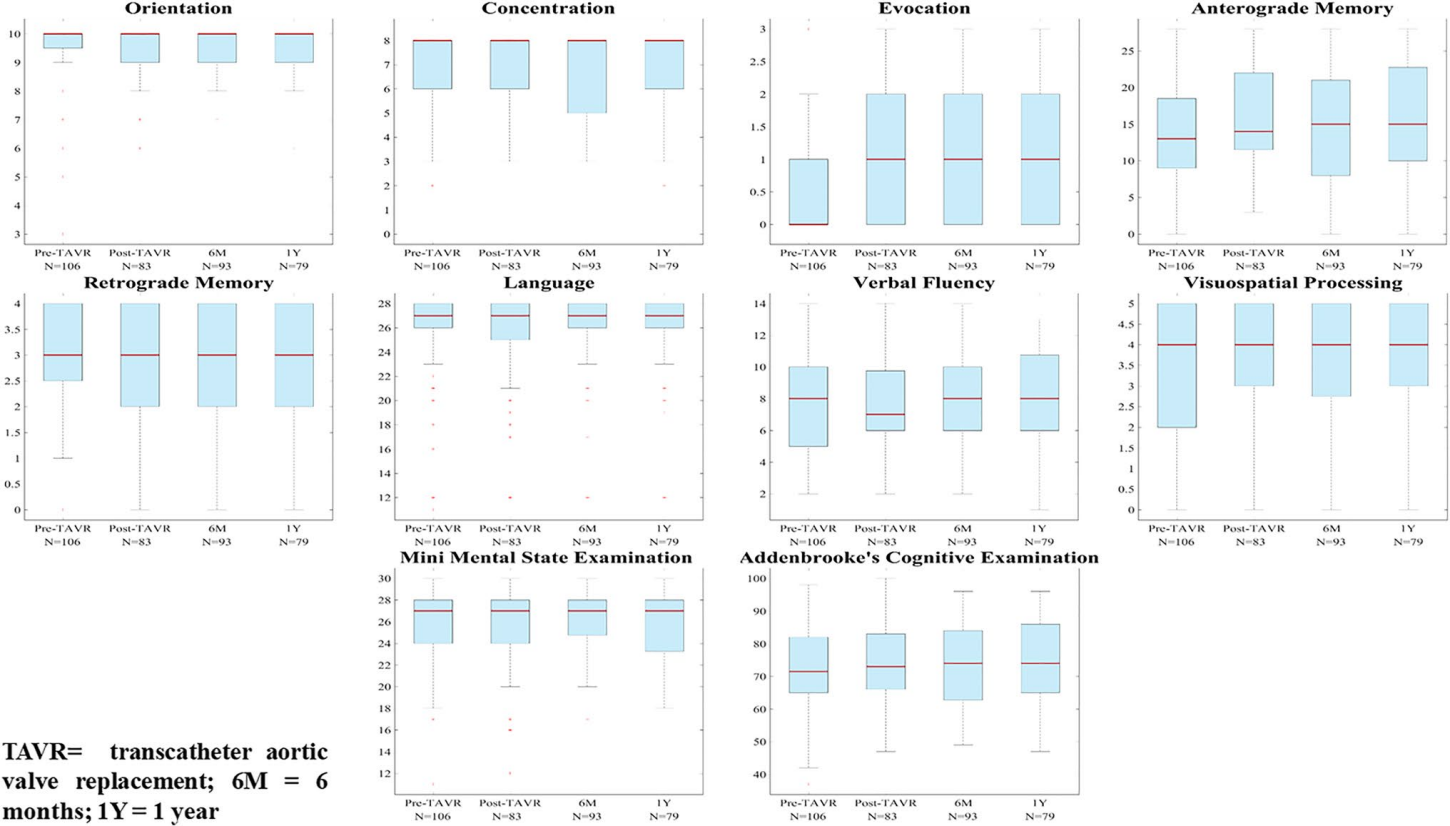
Box plots of cognitive scores pre-TAVR, post-TAVR, at 6 months and 1 year. Red line = median; top of box = 25th percentile; bottom of box = 75th percentile; plotted whisker = the most extreme data value that is not an outlier; red cross = outlier

Although there was an improvement in 3 domains and the ACE score (Fig. 5), the repeated-measures LMM model revealed no significant change in cognitive function over 1 year. The pre-TAVR score had a significant effect on scores at any later time points (*p* < 0.0001), with the greater change (improvement) of ACE scores, the lower was the pre-TAVR score. In ILL group III there was a significant decline in retrograde memory (*p* = 0.0483) and visual scores (*p* = 0.0151) compared to ILL groups I + II (Table 4). Apart from the improvement of evocation scores from pre-TAVR to post-TAVR, the Wilcoxon signed-rank test showed no significant change, in particular deterioration, regarding any domains across any different time points.

**Table 4:** Trend analysis of cognitive scores over time

**Outcome**	**Variables**	Regression coefficient (β)	95% CI	***p***
**ACE score** at different timepoints	Intercept	15.3131	4.7209; 25.9053	0.0052
	Pre-TAVR score	0.8184	0.6748; 0.9621	** < 0.0001**
	Time from TAVR	− 0.0358	− 0.2508; 0.1792	0.7411
**Change of ACE score**	Intercept	15.6595	4.9801; 26.3388	0.0046
	Pre-TAVR score	− 0.1844	− 0.3292; − 0.0395	**0.0137**
	Time from TAVR	− 0.0766	− 0.2829; 0.1298	0.4618
Effect of ILL on retrograde memory	Intercept	0.7391	0.0255; 1.4527	0.0425
	pre-TAVR score	0.7499	0.5465; 0.9533	** < 0.0001**
	ILL	− 0.4175	− 0.8328; − 0.0021	**0.0489**
	Time from TAVR	− 0.0056	− 0.0362; 0.0250	0.7189
Effect of ILL on visuospatial processing	Intercept	1.6757	1.0666; 2.2848	< 0.0001
	Pre-TAVR score	0.5988	0.4433; 0.7542	** < 0.0001**
	ILL	− 0.5514	− 0.9914; − 0.1113	**0.0151**
	Time from TAVR	− 0.0039	− 0.0360; 0.0281	0.8071

*ACE*, Addenbrooke’s cognitive examination; *ILL*, ischemic lesion load; *TAVR*, transcatheter aortic valve replacement

### Correlation of DTI metrics and cognitive output

Low diffusivities showed a trend towards higher cognitive scores in many ROIs but in most cases, it did not reach statistical significance after correction of the *p*-value for multiple comparisons. However, the negative correlation remained significant in some instances: in the left cingulate gyrus (association of low RD values and high language scores: *p* = 0.0012; low RD and high verbal fluency scores: *p* = 0.007) and in the genu (low AD and high retrograde memory scores: *p* = 0.0034).

## Discussion

Our main findings were (i) the significant reduction of diffusivities in 5 out of 7 WM ROIs between the post-TAVR and 6M DTI, (ii) the significant association of female sex with metric changes in 3 out of 7 ROIs and (iii) the significiant negative association of ILL and the revealed microstructural changes. We also found higher than reported incidence of IBLs (95% vs 84%) following TAVR, which might be explained by the higher sensitivity of the applied diffusion MRI acquisition thanks to its the greater number of encoding directions and therefore higher signal-to-noise and contrast-to-noise ratios relative to the widely used diffusion-weighted imaging sequence. To our knowledge, this is the first study to assess WM integrity with DTI in TAVR patients and correlate these findings with postprocedural IBLs and cognitive performance.

In numerous reports, the increased AD was interpreted as a sign of loss in fibre integrity linked to ageing [10, 14], age-related cognitive decline [8, 9]], cognitive impairment in Alzheimer’s disease [16], Parkinson’s disease [17] and multiple sclerosis [19]. The increase of AD might be explained by long-standing axonal damage, and as a consequence, a drop of restriction to water diffusion parallel to axons. This results also in increased MD. In conjunction with AD, but to a lesser extent, RD increases and diffusion becomes more isotropic, such as observed in chronic cerebral ischemia [6]. With regard to the specific anatomical structures assessed by us, significant increase of AD was reported in the genu of the corpus callosum in Parkinson’s disease versus controls [17], in the body of corpus callosum in elderly versus young subjects [10], and demented patients with Parkinson’s disease versus those without congitive impairment [17] in conjunction with significantly increased MD in the latter two examples. In our elderly TAVR candidates with visible chronic small vessel disease in the majority of cases [24], we might assume an a priori increase in diffusivities in line with the literature. With the improved cardiac output and cerebral perfusion following TAVR [47, 48], preexisting microstructural alterations can be potentially reversed, leading to quasi normalisation of the abnormally increased AD (resulting in net decrease of AD as shown by us in genu and right parahippocampal cingulum) or AD + MD (net decrease of AD + MD as detected in the body of the corpus callosum). Similarly, to our findings, significant decrease of AD [49] and significant decrease of AD + FA [50] was reported in the corticospinal tract of patients with idiopathic normal pressure hydrocephalus, who underwent cerebrospinal fluid derivation [49, 50] and were responders [50]. It is noteworthy that hydrocephalus was not a characteristic feature in our cohort as opposed to theirs; however, both patient populations are elderly, sharing high cardiovascular risks [51] and related ischemic WM damage. Another explanation of decreased AD (with or without MD reduction) can be the lower extracellular volume fraction (i.e. reduction of subtle otherwise not detectable vasogenic WM oedema), the higher membrane density and the increased axonal volume fraction [52] within the salvaged axons. In WM injury with predominant myelin degeneration, AD is not or only marginally affected. On the other hand, RD increases, reflecting a greater freedom of water motion perpendicular to axons [5].

In previous studies, significant increase of MD and RD was reported in the cingulate gyri in Alzheimer’s disease relative to healthy elderly adults [16] and in part of the cingulate gyri of older versus young participants [10]. Significantly higher RD was reported in the cingulate bundles of older versus young subjects in a further investigation, and the results showed that RD is a better predictor of cognitive performance in older adults [53]. In our study, the significantly reduced MD and RD found in the left cingulate gyrus show an opposite trend to the previous reports and might reflect reorganisation of the injured myelin. A statistically significant reduction in MD and RD of the whole brain and a statistically significant increase of the MMSE scores was observed 12 months following uncomplicated carotid endarterectomy in a small cohort [54], which might support our findings in the left cingulate gyrus.

The significant reduction of FA and a less marked but still significant reduction of AD with a subtle increase of RD demonstrated in the splenium might be related to WM degradation not relieved by TAVR. These changes may be indicative of ongoing ischemic WM damage on the microscopic level with irreversible axonal loss and reactive gliosis [3].

Some of the DTI metric changes seemed to be more pronounced in women of our cohort. Significantly greater decrease of AD and MD in the body of corpus callosum and right cingulate gyrus and significantly greater reduction of the AD in the left cingulate gyrus were found together with considerable gender differences in cardiovascular risks (higher incidence of diabetes mellitus in males) and comorbidities (higher frequence of previous myocardial infarction in males). As to whether DTI changes may be attributable directly to the gender or these gender-specific differences account for the alterations of the DTI metrics, we can not state with certainty. Based on the differences in cardiovascular risks and comorbidities, we could expect more advanced WM damage, therefore less microstructural reserve in men of our cohort, and conversely better outcomes in women. However, this explanation remains theoretical and elusive, as the small study cohort does not allow us to draw conclusions on casual relationships between differences in cardiovascular risks and change in diffusivities.

Similar gender characteristics to ours (difference in diabetes mellitus and myocardial infarction) were shown in a large TAVR population, where the short-term outcomes were not different in males versus females as opposed to the significantly worse all-cause mortality in males detected after a year [55].

Regarding post-TAVR ischemic injury, significant effect of ILL on diffusivity metric changes was revealed in 3 of our 7 ROIs as displayed in Fig. 4. In the body of the corpus callosum, all diffusivities (AD, MD, RD) showed a significant negative association with ILL. The most marked difference was found between the decrease of AD and MD in ILL groups I–II and the increase of AD and MD in ILL group III. The increase of RD and the decrease of FA in group III are in contrast to the reduction of RD in groups I–II and increase of FA in group II, however, in case of FA without reaching the threshold of significance. In other words, the higher ILL was associated with unfavourable microstructural changes, i.e. overall increase in diffusivities and some loss of diffusion directionality. Moreover, in the left cingulate gyrus, significant difference in MD reduction was found between the ILL group II and group III. The decrease of MD was more prominent in the group with intermediate ILL relative to the highest ILL. The less pronounced (non significant) decrease of FA in the ILL group III versus the increase of FA in groups I-II can also be connected to the lack of WM reorganisation in case of larger ischemic load. In the splenium of the corpus callosum, where the DTI metric alterations indicated continuing overall WM degeneration, the same effect of ILL was shown across the different groups. The reduction of MD in ILL group I was in contrast to the slightly increased MD in group III, although without a significant difference following Bonferroni correction. The increase in MD and the smaller reduction of AD in ILL group III versus the more marked reduction of AD and MD in groups I-II are in accordance with our findings outlined above, all suggesting that white matter integrity loss is less likely to be reversed with higher ILL.

Functional meaningfulness of the revealed microstructural differences was supported by the mild improvement in some of the cognitive domains and the stability of overall cognitive function despite the frequent post-TAVR ischemic brain injury in this eldely, frail and comorbid patient cohort. In the Spearman correlation test, low diffusivity values showed a trend towards higher cognitive scores in many ROIs but in most cases without reaching statistical significance. However, the negative correlation remained significant for the association of RD and language/verbal fluency in the left cingulate gyrus, and that of AD and retrograde memory in the genu. Our results need to be interpreted with caution as we did not evaluate all white matter tracts and the role of the studied anatomical structures in a specific cognitive performance is more complex and beyond the scopes of the present study.

The importance of TAVR-related cerebral ischemic injury is also highlighted by the fact that higher ILL showed a significant negative association with retrograde memory and visual performance. Our results were in accordance with those of a meta-analysis showing a significant positive relationship between post-TAVR cognitive dysfunction and the number of silent brain infarcts [40]. However, it should be noted that the aetiology of IBL is diverse including many patient-specific (atrial fibrillation, heart failure, vascular disease) and procedure-specific factors (pre-dilatation and valve positioning) explaining the non-consistent association with post-procedural cognitive changes reported in the literature [37]. Further studies on the long-term effect of IBLs on cognition are warranted, especially with the extension of TAVR indication to younger patients with good baseline cognition.

The limitations of our investigation are sixfold. (1) DTI is a mathematical representation of the underlying structure not always reflecting true brain anatomy. Noise, partial volume effects and crossing fibres within a voxel can result in false positive/negative results. (2) The interpretation of DTI indices and their relation to pathological processes are somewhat theoretical. Histological data would have allowed for a more direct link between diffusivity changes and microstructural alterations. (3) Further limitation is that we did not study all white matter tracts, only those with reliable manual correction of the automated segmentation. (4) We did not analyse the effect of chronic cerebral microbleeds, anticoagulant and/or antiplatelet therapy either on DTI indices or on cognition — these aims were beyond the scope of our study. (5) The fact that patients with an improved general and cognitive state are more likely to turn up and participate at follow-ups might have led to selection bias and thus caused underestimation of cognitive decline. (6) Finally a larger sample size might have provided us with more statistical power.

Diffusion properties and their change following TAVR have not been studied by far; moreover, ageing and most cited pathological processes and their effects on cerebral diffusion cannot be reversed. The interpretation of reduced AD, MD and RD is somewhat elusive as we could only rely on the fact, that our findings are opposite to numerous studies of neurodegenerative processes/ageing. We do not yet have conclusive evidence that our findings reflect true microstructural improvement directly attributable to TAVR. Our results will need to be interpreted with caution, as a mixture of a decrease in diffusivities caused by the clearance of subtle oedema with or without reorganisation of myelin and/or an increase caused by on-going ischemic WM degeneration.

In conclusion, significant effect of TAVR on cerebral microstructural properties was found with reduced diffusivities opposite to the general trends reported in studies of various neurodegenerative conditions and ageing, notably in women (in line with the lower cardiovascular risk revealed in females of our cohort) and lower ILL. Moreover, the overall cognitive function was maintained despite the high intrinsic ischemic load following TAVR with significant inverse relationship between ILL and cognitive scores in some domains.

## Data, materials and/or code availability.

The datasets generated and analysed during the current study are available from the corresponding author on reasonable request.

## Supplementary Information

Below is the link to the electronic supplementary material.

Supplementary file1 (DOCX 62 KB)

## Abbreviations

ACE: Addenbrooke’s cognitive examination
ANOVA: Analysis of variance
AS: Aortic stenosis
AD: Axial diffusivity
DTI: Diffusion tensor imaging
FA: Fractional anisotropy
IBL: Ischemic brain lesions
ILL: Ischemic lesion load
LMM: Linear mixed-effect model
MD: Mean diffusivity
MMSE: Mini mental state examination
RD: Radial diffusivity
ROI: Region of interest
TAVR: Transcatheter aortic valve replacement
WM: White matter
