# Supplementary material for: Microstructural alterations measured by diffusion tensor imaging following transcatheter aortic valve replacement and their association with cerebral ischemic injury and cognitive function — a prospective study
Source: Neuroradiology. 2022 Aug 1;64(12):2343–56. doi: 10.1007/s00234-022-03017-5 (PMC9643192; doi:10.1007/s00234-022-03017-5)
Supplement: Supplementary file 1 — Supplementary file1 (DOCX 62 KB) [file 234_2022_3017_MOESM1_ESM.docx]

**Supplement Table 1.** Results of repeated measures analysis of variance on effects of sex, age and ischemic lesion load on diffusion tensor imaging metric changes

| Repeated measures modell covariants: (Intercept), Sex female, Age, ILL group I, ILL group II | | |
| --- | --- | --- |
| **BODY OF CORPUS CALLOSUM** | | |
| ***FA*** | | |
|  | F | p-value^*^ |
| (Intercept):Time | 1,0178 | 0,3164 |
| Sex:Time | 0,1784 | 0,6740 |
| Age:Time | 1,2683 | 0,2638 |
| ILL group:Time | 1,2427 | 0,2947 |
| Error(Time) | 1,0000 | 0,5000 |
|  |  |  |
| Post hoc analysis (ILL group): |  | p-value^**^ |
| I - II |  | 0,9991 |
| I - III |  | 0,9836 |
| II - III |  | 0,9785 |
| ***MD*** | | |
|  | F | p-value^*^ |
| (Intercept):Time | 0,0253 | 0,8742 |
| Sex:Time | **5,2154** | **0,0254** |
| Age:Time | 0,1947 | 0,6603 |
| ILL_group:Time | **5,7180** | **0,0050** |
| Error(Time) | 1,0000 | 0,5000 |
|  |  |  |
| Post hoc analysis (ILL group): |  | p-value^**^ |
| I - II |  | 0,7509 |
| I - III |  | 0,0457 |
| II - III |  | 0,2601 |
| ***AD*** | | |
|  | F | p-value^*^ |
| (Intercept):Time | 0,5819 | 0,4481 |
| Sex:Time | **7,8653** | **0,0065** |
| Age:Time | 1,5269 | 0,2206 |
| ILL_group:Time | **5,8505** | **0,0045** |
| Error(Time) | 1,0000 | 0,5000 |
|  |  |  |
| Post hoc analysis (ILL group): |  | p-value^**^ |
| I - II |  | 0,6973 |
| I - III |  | 0,0183 |
| II - III |  | 0,1662 |
| ***RD*** | | |
|  | F | p-value^*^ |
| (Intercept):Time | 0,0380 | 0,8461 |
| Sex:Time | 2,8593 | 0,0952 |
| Age:Time | 0,0030 | 0,9562 |
| ILL_group:Time | **4,2827** | **0,0175** |
| Error(Time) | 1,0000 | 0,5000 |
|  |  |  |
| Post hoc analysis (ILL group): |  | p-value^**^ |
| I - II |  | 0,8963 |
| I - III |  | 0,3167 |
| II - III |  | 0,6183 |
| **CINGULATE GYRUS RIGHT** | | |
| ***FA*** | | |
|  | F | p-value^*^ |
| (Intercept):Time | 0,1624 | 0,6881 |
| Sex:Time | 2,8449 | 0,0960 |
| Age:Time | 0,0979 | 0,7553 |
| ILL_group:Time | 1,1007 | 0,3382 |
| Error(Time) | 1,0000 | 0,5000 |
|  |  |  |
| Post hoc analysis (ILL group): |  | p-value^**^ |
| I - II |  | 0,6935 |
| I - III |  | 0,7418 |
| II - III |  | 0,9960 |
| ***MD*** | | |
|  | F | p-value^*^ |
| (Intercept):Time | 1,0753 | 0,3032 |
| Sex:Time | **9,1228** | **0,0035** |
| Age:Time | 0,5434 | 0,4634 |
| ILL_group:Time | 1,1831 | 0,3122 |
| Error(Time) | 1,0000 | 0,5000 |
|  |  |  |
| Post hoc analysis (ILL group): |  | p-value^**^ |
| I - II |  | 0,5721 |
| I - III |  | 0,3976 |
| II - III |  | 0,0832 |
| ***AD*** | | |
|  | F | p-value^*^ |
| (Intercept):Time | 0,8880 | 0,3492 |
| Sex:Time | **16,7079** | **0,0001** |
| Age:Time | 0,5556 | 0,4585 |
| ILL_group:Time | 2,9204 | 0,0603 |
| Error(Time) | 1,0000 | 0,5000 |
|  |  |  |
| Post hoc analysis (ILL group): |  | p-value^**^ |
| I - II |  | 0,3155 |
| I - III |  | 0,9565 |
| II - III |  | 0,2332 |
| ***RD*** | | |
|  | F | p-value^*^ |
| (Intercept):Time | 0,3923 | 0,5330 |
| Sex:Time | 0,5624 | 0,4557 |
| Age:Time | 0,1481 | 0,7015 |
| ILL_group:Time | 0,6144 | 0,5438 |
| Error(Time) | 1,0000 | 0,5000 |
|  |  |  |
| Post hoc analysis (ILL group): |  | p-value^**^ |
| I - II |  | 0,9925 |
| I - III |  | 0,3557 |
| II - III |  | 0,4707 |
| **CINGULATE GYRUS LEFT** | | |

| ***FA*** | | |
| --- | --- | --- |
|  | F | p-value^*^ |
| (Intercept):Time | 3,9937 | 0,0494 |
| Sex:Time | 2,6541 | 0,1076 |
| Age:Time | 3,6973 | 0,0585 |
| ILL_group:Time | 0,4648 | 0,6301 |
| Error(Time) | 1,0000 | 0,5000 |
|  |  |  |
| Post hoc analysis (ILL group): |  | p-value^**^ |
| I - II |  | 0,9609 |
| I - III |  | 0,8884 |
| II - III |  | 0,9827 |
| ***MD*** | | |
|  | F | p-value^*^ |
| (Intercept):Time | 0,0001 | 0,9923 |
| Sex:Time | 3,2490 | 0,0757 |
| Age:Time | 0,0974 | 0,7559 |
| ILL_group:Time | 0,9744 | 0,3823 |
| Error(Time) | 1,0000 | 0,5000 |
|  |  |  |
| Post hoc analysis (ILL group): |  | p-value^**^ |
| I - II |  | 0,3121 |
| I - III |  | 0,1790 |
| II - III |  | **0,0087** |
| ***AD*** | | |
|  | F | p-value^*^ |
| (Intercept):Time | 1,4348 | 0,2349 |
| Sex:Time | **7,9737** | **0,0062** |
| Age:Time | 1,9016 | 0,1722 |
| ILL_group:Time | 1,6466 | 0,1999 |
| Error(Time) | 1,0000 | 0,5000 |
|  |  |  |
| Post hoc analysis (ILL group): |  | p-value^**^ |
| I - II |  | 0,4454 |
| I - III |  | 0,8015 |
| II - III |  | 0,1999 |
|  |  |  |
| ***RD*** | | |
|  | F | p-value^*^ |
| (Intercept):Time | 1,1942 | 0,2781 |
| Sex:Time | 0,0141 | 0,9059 |
| Age:Time | 0,7101 | 0,4022 |
| ILL_group:Time | 0,2506 | 0,7790 |
| Error(Time) | 1,0000 | 0,5000 |
|  |  |  |
| Post hoc analysis (ILL group): |  | p-value^**^ |
| I - II |  | 0,8536 |
| I - III |  | 0,2971 |
| II - III |  | 0,1447 |
| **PARAHIPPOCAMPAL CINGULUM RIGHT** | | |
| ***FA*** | | |
|  | F | p-value^*^ |
| (Intercept):Time | 0,0008 | 0,9773 |
| Sex:Time | 0,8925 | 0,3480 |
| Age:Time | 0,0031 | 0,9558 |
| ILL_group:Time | 0,3322 | 0,7184 |
| Error(Time) | 1,0000 | 0,5000 |
|  |  |  |
| Post hoc analysis (ILL group): |  | p-value^**^ |
| I - II |  | 0,7997 |
| I - III |  | 0,7695 |
| II - III |  | 0,9991 |
| ***MD*** | | |
|  | F | p-value^*^ |
| (Intercept):Time | 1,7424 | 0,1910 |
| Sex:Time | 0,1480 | 0,7015 |
| Age:Time | 2,2941 | 0,1342 |
| ILL_group:Time | 0,1328 | 0,8758 |
| Error(Time) | 1,0000 | 0,5000 |
|  |  |  |
| Post hoc analysis (ILL group): |  | p-value^**^ |
| I - II |  | 0,6278 |
| I - III |  | 0,3976 |
| II - III |  | 0,9373 |
|  |  |  |
| ***AD*** | | |
|  | F | p-value^*^ |
| (Intercept):Time | 2,0353 | 0,1580 |
| Sex:Time | 0,9604 | 0,3304 |
| Age:Time | 2,7919 | 0,0991 |
| ILL_group:Time | 0,0755 | 0,9273 |
| Error(Time) | 1,0000 | 0,5000 |
|  |  |  |
| Post hoc analysis (ILL group): |  | p-value^**^ |
| I - II |  | 0,8251 |
| I - III |  | 0,6110 |
| II - III |  | 0,9433 |
| ***RD*** | | |
|  | F | p-value^*^ |
| (Intercept):Time | 1,3039 | 0,2573 |
| Sex:Time | 0,0001 | 0,9911 |
| Age:Time | 1,6630 | 0,2013 |
| ILL_group:Time | 0,1850 | 0,8315 |
| Error(Time) | 1,0000 | 0,5000 |
|  |  |  |
| Post hoc analysis (ILL group): |  | p-value^**^ |
| I - II |  | 0,6372 |
| I - III |  | 0,4488 |
| II - III |  | 0,9597 |
| **PARAHIPPOCAMPAL CINGULUM LEFT** | | |
| ***FA*** | | |
|  | F | p-value^*^ |
| (Intercept):Time | 1,9876 | 0,1629 |
| Sex:Time | 0,4981 | 0,4826 |
| Age:Time | 2,1288 | 0,1489 |
| ILL_group:Time | 2,7031 | 0,0738 |
| Error(Time) | 1,0000 | 0,5000 |
|  | 1,9876 | 0,1629 |
|  |  |  |
| Post hoc analysis (ILL group): |  | p-value^**^ |
| I - II |  | 0,9767 |
| I - III |  | 0,6799 |
| II -III |  | 0,5932 |
| ***MD*** | | |
|  | F | p-value^*^ |
| (Intercept):Time | 0,7559 | 0,3875 |
| Sex:Time | 2,8307 | 0,0968 |
| Age:Time | 0,6430 | 0,4253 |
| ILL_group:Time | 0,9344 | 0,3975 |
| Error(Time) | 1,0000 | 0,5000 |
|  |  |  |
| Post hoc analysis: |  |  |
| ILL_group |  | p-value^**^ |
| I - II |  | 0,9202 |
| I - III |  | 0,9990 |
| II -III |  | 0,9128 |
| ***AD*** | | |
|  | F | p-value^*^ |
| (Intercept):Time | 0,0464 | 0,8301 |
| Sex:Time | 1,2850 | 0,2607 |
| Age:Time | 0,0127 | 0,9105 |
| ILL_group:Time | 0,0984 | 0,9064 |
| Error(Time) | 1,0000 | 0,5000 |
|  |  |  |
| Post hoc analysis (ILL group): |  | p-value^**^ |
| I - II |  | 0,9859 |
| I - III |  | 0,9405 |
| II - III |  | 0,9862 |
| ***RD*** | | |
|  | F | p-value^*^ |
| (Intercept):Time | 1,4764 | 0,2283 |
| Sex:Time | 3,3644 | 0,0708 |
| Age:Time | 1,3985 | 0,2409 |
| ILL_group:Time | 1,7538 | 0,1804 |
| Error(Time) | 1,0000 | 0,5000 |
|  |  |  |
| Post hoc analysis (ILL group): |  | p-value^**^ |
| I - II |  | 0,8887 |
| I - III |  | 0,9382 |
| II - III |  | 0,7316 |
| **GENU OF CORPUS CALLOSUM** | | |
| ***FA*** | | |
|  | F | p-value^*^ |
| (Intercept):Time | 0,5981 | 0,4418 |
| Sex:Time | 0,4440 | 0,5073 |
| Age:Time | 0,8617 | 0,3564 |
| ILL_group:Time | 1,4027 | 0,2526 |
| Error(Time) | 1,0000 | 0,5000 |
|  |  |  |
| Post hoc analysis (ILL group): |  | p-value^**^ |
| I - II |  | 0,4764 |
| I - III |  | 0,6600 |
| II - III |  | 0,9542 |
| ***MD*** | | |
|  | F | p-value^*^ |
| (Intercept):Time | 0,1937 | 0,6612 |
| Sex:Time | 1,4284 | 0,2359 |
| Age:Time | 0,3453 | 0,5586 |
| ILL_group:Time | 0,6402 | 0,5302 |
| Error(Time) | 1,0000 | 0,5000 |
|  |  |  |
| Post hoc analysis (ILL group): |  | p-value^**^ |
| I - II |  | 0,9115 |
| I - III |  | 0,8223 |
| II - III |  | 0,6146 |
| ***AD*** | | |
|  | F | p-value^*^ |
| (Intercept):Time | 0,6010 | 0,4407 |
| Sex:Time | 1,9831 | 0,1634 |
| Age:Time | 1,1466 | 0,2878 |
| ILL_group:Time | 1,0326 | 0,3613 |
| Error(Time) | 1,0000 | 0,5000 |
|  |  |  |
| Post hoc analysis (ILL group): |  | p-value^**^ |
| ILL_group |  | p-value^**^ |
| I - II |  | 0,8927 |
| I - III |  | 0,4006 |
| II -III |  | 0,7162 |
| ***RD*** | | |
|  | F | p-value^*^ |
| (Intercept):Time | 0,0253 | 0,8739 |
| Sex:Time | 0,7564 | 0,3873 |
| Age:Time | 0,0361 | 0,8498 |
| ILL_group:Time | 0,8269 | 0,4415 |
| Error(Time) | 1,0000 | 0,5000 |
|  |  |  |
| Post hoc analysis (ILL group): |  | p-value^**^ |
| I - II |  | 0,5728 |
| I - III |  | 0,9697 |
| II - III |  | 0,7437 |
| **SPLENIUM OF CORPUS CALLOSUM** | | |
| ***FA*** | | |
|  | F | p-value^*^ |
| (Intercept):Time | 0,2723 | 0,6034 |
| Sex:Time | 1,9443 | 0,1675 |
| Age:Time | 0,6844 | 0,4108 |
| ILL_group:Time | 0,0519 | 0,9495 |
| Error(Time) | 1,0000 | 0,5000 |
|  |  |  |
| Post hoc analysis (ILL group): |  | p-value^**^ |
| I - II |  | 0,9445 |
| I - III |  | 0,8838 |
| II -III |  | 0,7372 |
| ***MD*** | | |
|  | F | p-value^*^ |
| (Intercept):Time | 0,7679 | 0,3838 |
| Sex:Time | 0,0352 | 0,8518 |
| Age:Time | 0,7272 | 0,3966 |
| ILL_group:Time | 0,4999 | 0,6087 |
| Error(Time) | 1,0000 | 0,5000 |
|  |  |  |
| Post hoc analysis (ILL group): |  | p-value^**^ |
| I - II |  | 0,9811 |
| I - III |  | 0,1063 |
| II -III |  | 0,0986 |
| ***AD*** | | |
|  | F | p-value^*^ |
| (Intercept):Time | 2,8627 | 0,0950 |
| Sex:Time | 0,2242 | 0,6373 |
| Age:Time | 3,7012 | 0,0583 |
| ILL_group:Time | 1,5172 | 0,2263 |
| Error(Time) | 1,0000 | 0,5000 |
|  |  |  |
| Post hoc analysis (ILL group): |  | p-value^**^ |
| I - II |  | 0,9788 |
| I - III |  | 0,0462 |
| II -III |  | 0,1023 |
| ***RD*** | | |
|  | F | p-value^*^ |
| (Intercept):Time | 0,0999 | 0,7528 |
| Sex:Time | 0,2744 | 0,6020 |
| Age:Time | 0,0244 | 0,8763 |
| ILL_group:Time | 0,1275 | 0,8805 |
| Error(Time) | 1,0000 | 0,5000 |
|  |  |  |
| Post hoc analysis (ILL group): |  | p-value^**^ |
| I - II |  | 0,9183 |
| I - III |  | 0,3766 |
| II -III |  | 0,2427 |

AD= axial diffusivity; F= F-statistic; FA= fractional anisotropy; FU= follow up; ILL= ischemic lesion load; MD= mean diffusivity; RD= radial diffusivity.

^*^P-values with Greenhouse-Geisser, Huynh-Feldt and lower bound adjustment (not included) were similar to p-values for the corresponding F-statistics in all categories. **Bonferroni-corrected p-values significant if <0.0167.
